# 2,5-Bis(4-fluoro­phen­yl)-2-methyl­sulfanyl-1-benzofuran

**DOI:** 10.1107/S1600536811007458

**Published:** 2011-03-05

**Authors:** Hong Dae Choi, Pil Ja Seo, Byeng Wha Son, Uk Lee

**Affiliations:** a Department of Chemistry, Dongeui University, San 24 Kaya-dong Busanjin-gu, Busan 614-714, Republic of Korea; b Department of Chemistry, Pukyong National University, 599-1 Daeyeon 3-dong, Nam-gu, Busan 608-737, Republic of Korea

## Abstract

The crystal studied of the title compound, C_21_H_14_F_2_OS, was an inversion twin with a 0.67 (8):0.33 (8) domain ratio. The 4-fluoro­phenyl ring in the 2-position makes a dihedral angle of 25.14 (6)° with the mean plane of the benzofuran fragment, and the dihedral angle between 4-fluoro­phenyl ring in the 5-position and the mean plane of the benzofuran fragment is 28.50 (7)°. In the crystal, mol­ecules are linked through weak inter­molecular C—H⋯F and C—H⋯π inter­actions.

## Related literature

For the pharmacological activity of benzofuran compounds, see: Aslam *et al.* (2006 ▶); Galal *et al.* (2009 ▶); Khan *et al.* (2005 ▶). For natural products with benzofuran rings, see: Akgul & Anil (2003 ▶); Soekamto *et al.* (2003 ▶). For structural studies of related 3-alkyl­sulfanyl-2-(4-fluoro­phen­yl)-5-phenyl-1-benzofuran derivatives, see: Choi *et al.* (2009 ▶, 2010 ▶). 
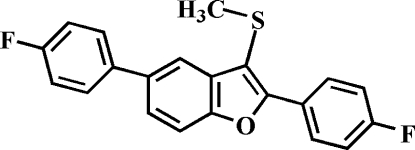


## Experimental

### 

#### Crystal data

C_21_H_14_F_2_OS
*M* *_r_* = 352.38
Monoclinic, 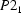

*a* = 10.7673 (7) Å
*b* = 7.2986 (5) Å
*c* = 11.5145 (8) Å
β = 116.124 (1)°
*V* = 812.44 (10) Å^3^
*Z* = 2
Mo *K*α radiation
μ = 0.23 mm^−1^
*T* = 173 K
0.32 × 0.29 × 0.13 mm

#### Data collection

Bruker SMART APEXII CCD diffractometer
Absorption correction: multi-scan (*SADABS*; Bruker, 2009 ▶) *T*_min_ = 0.930, *T*_max_ = 0.972
7891 measured reflections
3710 independent reflections
3501 reflections with *I* > 2σ(*I*)
*R*_int_ = 0.023

#### Refinement

*R*[*F* ^2^ > 2σ(*F* ^2^)] = 0.038
*wR*(*F* ^2^) = 0.101
*S* = 1.06
3710 reflections
228 parameters
1 restraint
H-atom parameters constrained
Δρ_max_ = 0.33 e Å^−3^
Δρ_min_ = −0.21 e Å^−3^
Absolute structure: Flack (1983 ▶), 1700 Friedel pairs
Flack parameter: 0.33 (8)

### 

Data collection: *APEX2* (Bruker, 2009 ▶); cell refinement: *SAINT* (Bruker, 2009 ▶); data reduction: *SAINT*; program(s) used to solve structure: *SHELXS97* (Sheldrick, 2008 ▶); program(s) used to refine structure: *SHELXL97* (Sheldrick, 2008 ▶); molecular graphics: *ORTEP-3* (Farrugia, 1997 ▶) and *DIAMOND* (Brandenburg, 1998 ▶); software used to prepare material for publication: *SHELXL97*.

## Supplementary Material

Crystal structure: contains datablocks global, I. DOI: 10.1107/S1600536811007458/sj5110sup1.cif

Structure factors: contains datablocks I. DOI: 10.1107/S1600536811007458/sj5110Isup2.hkl

Additional supplementary materials:  crystallographic information; 3D view; checkCIF report

## Figures and Tables

**Table 1:** Hydrogen-bond geometry (Å, °) *Cg* is the centroid of the C15–C20 4-fluoro­phenyl ring.

*D*—H⋯*A*	*D*—H	H⋯*A*	*D*⋯*A*	*D*—H⋯*A*
C6—H6⋯F1^i^	0.95	2.45	3.322 (2)	153
C10—H10⋯*Cg*^ii^	0.95	2.67	3.441 (2)	139
C17—H17⋯*Cg*^iii^	0.95	2.86	3.557 (2)	131

Symmetry codes: (i) 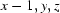; (ii) 
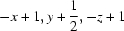
; (iii) 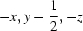.

**Fig. 1.**
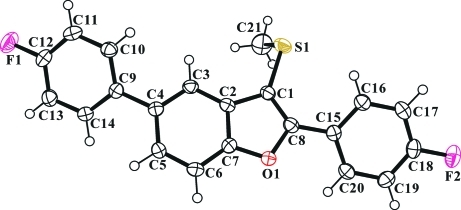
The molecular structure of the title compound with the atom numbering scheme. Displacement ellipsoids are drawn at the 30% probability level. H atoms are presented as a small spheres of arbitrary radius.

**Fig. 2.**
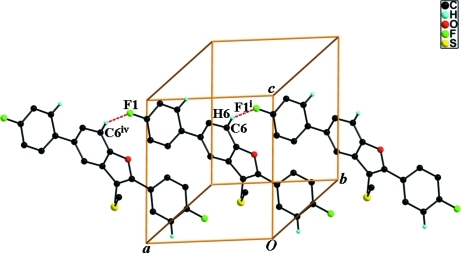
A view of the C—H···F interactions (dotted lines) in the crystal structure of the title compound. [Symmetry codes: (i) x - 1, y, z; (iv) x+ 1, y, z.]

**Fig. 3.**
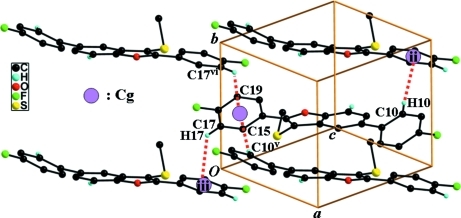
A view of the C—H···π interactions (dotted lines) in the crystal structure of the title compound. [Symmetry codes: (ii) - x + 1, y + 1/2, - z + 1; (iii) - x, y- 1/2, - z; (v)1 - x, y - 1/2, - z + 1; (vi) - x, y + 1/2, - z.]

## supplementary crystallographic information

### Comment

Many compounds having a benzofuran ring system exhibit interesting pharmacological properties such as antifungal, antitumor and antiviral, and antimicrobial activities (Aslam *et al.*, 2006, Galal *et al.*, 2009, Khan *et al.*, 2005). These compounds occur in a wide range of natural products (Akgul & Anil, 2003; Soekamto *et al.*, 2003). As a part of our ongoing study of substituent effects on the solid state structures of 3-alkylsulfanyl-2-(4-fluorophenyl)-5-phenyl-1-benzofuran analogues (Choi *et al.*, 2009, 2010), we report herein on the crystal structure of the title compound.

The title compound crystallizes as the non-centrosymmetric space group *P*2_1_ in spite of having no asymmetric C atoms. The crystal studied was an inversion twin with a 0.33 (8) : 0.67 (8) domain ratio.

In the title molecule (Fig. 1), the benzofuran unit is essentially planar, with a mean deviation of 0.009 (1) Å from the least-squares plane defined by the nine constituent atoms. In the crystal structure, the dihedral angle formed by the 4-fluorophenyl ring (C15–C20) and the mean plane of the benzofuran fragment is 25.14 (6)°, and the (C9–C14) 4-fluorophenyl ring makes a dihedral angle of 28.50 (7)° with the mean plane of the benzofuran fragment. The molecular packing (Fig. 2) is stabilized by weak intermolecular C—H···F hydrogen bonds between a benzene H atom and the F atom of the (C9–C14) 4-fluorophenyl ring (Table 1; C6—H6···F1^i^). The molecular packing (Fig. 3) also exhibits intermolecular C—H···π interactions; the first one between a 4-fluorophenyl H atom in the 5-position and the (C15–C20) 4-fluorophenyl ring  (Table 1; C10–H10···Cg^ii^), and the second one between the 4-fluorophenyl H atom in the 2-position and the (C15–C20) 4-fluorophenyl ring  (Table 1; C17—H17···Cg^iii^) (Cg is the centroid of the C15–C20 4-fluorophenyl ring).

### Experimental

Zinc chloride (245 mg, 1.8 mmol) was added to a stirred solution of 4-fluoro-4'-hydroxybiphenyl (339 mg, 1,8 mmol) and 2-chloro-4'-fluoro-2-methylsulfanylacetophenone (395 mg, 1.8 mmol) in dichloromethane (30 mL) at room temperature, and stirring was continued at the same temperature for 40 min. The reaction was quenched by the addition of water and the organic layer was separated, dried over magnesium sulfate, filtered and concentrated at reduced pressure. The residue was purified by column chromatography (carbon tetrachloride) to afford the title compound as a colorless solid [yield 65%, m.p. 438–439 K; Rf = 0.69 (carbon tetrachloride)]. Single crystals suitable for X-ray diffraction were prepared by slow evaporation of a solution of the title compound in acetone at room temperature.

### Refinement

The reported Flack parameter was obtained by TWIN/BASF procedure in SHELXL (Sheldrick, 2008). All H atoms were positioned geometrically and refined using a riding model, with C—H = 0.95 Å  for aryl  and 0.98 Å for methyl H atoms. *U*_iso_(H) =1.2*U*_eq_(C)  for aryl and 1.5*U*_eq_(C) for methyl H atoms.

### Crystal data

**Table:**

C_21_H_14_F_2_OS	*F*(000) = 364
*M**_r_* = 352.38	*D*_x_ = 1.440 Mg m^−^^3^
Monoclinic, *P*2_1_	Mo *K*α radiation, λ = 0.71073 Å
Hall symbol: P 2yb	Cell parameters from 3532 reflections
*a* = 10.7673 (7) Å	θ = 2.2–27.1°
*b* = 7.2986 (5) Å	µ = 0.23 mm^−^^1^
*c* = 11.5145 (8) Å	*T* = 173 K
β = 116.124 (1)°	Block, colourless
*V* = 812.44 (10) Å^3^	0.32 × 0.29 × 0.13 mm
*Z* = 2	

### Data collection

**Table:**

Bruker SMART APEXII CCD diffractometer	3710 independent reflections
Radiation source: rotating anode	3501 reflections with *I* > 2σ(*I*)
graphite multilayer	*R*_int_ = 0.023
Detector resolution: 10.0 pixels mm^-1^	θ_max_ = 27.5°, θ_min_ = 2.0°
φ and ω scans	*h* = −13→13
Absorption correction: multi-scan (*SADABS*; Bruker, 2009)	*k* = −9→9
*T*_min_ = 0.930, *T*_max_ = 0.972	*l* = −14→14
7891 measured reflections	

### Refinement

**Table:**

Refinement on *F*^2^	Secondary atom site location: difference Fourier map
Least-squares matrix: full	Hydrogen site location: difference Fourier map
*R*[*F*^2^ > 2σ(*F*^2^)] = 0.038	H-atom parameters constrained
*wR*(*F*^2^) = 0.101	*w* = 1/[σ^2^(*F*_o_^2^) + (0.056*P*)^2^ + 0.1486*P*] where *P* = (*F*_o_^2^ + 2*F*_c_^2^)/3
*S* = 1.06	(Δ/σ)_max_ < 0.001
3710 reflections	Δρ_max_ = 0.33 e Å^−^^3^
228 parameters	Δρ_min_ = −0.21 e Å^−^^3^
1 restraint	Absolute structure: Flack (1983), 1700 Friedel pairs
Primary atom site location: structure-invariant direct methods	Flack parameter: 0.33 (8)

### Special details

**Table:**

Geometry. All esds (except the esd in the dihedral angle between two l.s. planes) are estimated using the full covariance matrix. The cell esds are taken into account individually in the estimation of esds in distances, angles and torsion angles; correlations between esds in cell parameters are only used when they are defined by crystal symmetry. An approximate (isotropic) treatment of cell esds is used for estimating esds involving l.s. planes.
Refinement. Refinement of F^2^ against ALL reflections. The weighted R-factor wR and goodness of fit S are based on F^2^, conventional R-factors R are based on F, with F set to zero for negative F^2^. The threshold expression of F^2^ > 2sigma(F^2^) is used only for calculating R-factors(gt) etc. and is not relevant to the choice of reflections for refinement. R-factors based on F^2^ are statistically about twice as large as those based on F, and R- factors based on ALL data will be even larger.

### Fractional atomic coordinates and isotropic or equivalent isotropic displacement parameters (Å^2^)

**Table:**

	*x*	*y*	*z*	*U*_iso_*/*U*_eq_	
S1	0.43705 (5)	0.35582 (9)	0.12177 (4)	0.03705 (15)	
F1	1.36155 (13)	0.4416 (2)	0.73988 (15)	0.0491 (4)	
F2	−0.24680 (12)	0.4131 (2)	−0.00514 (13)	0.0463 (3)	
O1	0.36309 (13)	0.4157 (2)	0.42787 (12)	0.0294 (3)	
C1	0.43510 (19)	0.3862 (3)	0.27194 (17)	0.0269 (4)	
C2	0.55585 (18)	0.3959 (3)	0.39519 (16)	0.0253 (4)	
C3	0.69845 (18)	0.3930 (3)	0.43512 (17)	0.0260 (4)	
H3	0.7346	0.3790	0.3738	0.031*	
C4	0.78673 (18)	0.4111 (3)	0.56623 (17)	0.0243 (4)	
C5	0.7295 (2)	0.4268 (3)	0.65587 (18)	0.0280 (4)	
H5	0.7905	0.4363	0.7454	0.034*	
C6	0.5895 (2)	0.4287 (3)	0.61797 (18)	0.0303 (4)	
H6	0.5523	0.4395	0.6788	0.036*	
C7	0.5052 (2)	0.4142 (3)	0.48647 (18)	0.0277 (4)	
C8	0.32360 (19)	0.3989 (3)	0.29668 (17)	0.0273 (4)	
C9	0.93978 (18)	0.4152 (3)	0.61213 (16)	0.0243 (4)	
C10	1.0238 (2)	0.5099 (3)	0.72483 (19)	0.0290 (4)	
H10	0.9826	0.5693	0.7726	0.035*	
C11	1.1652 (2)	0.5193 (3)	0.7686 (2)	0.0344 (5)	
H11	1.2214	0.5846	0.8453	0.041*	
C12	1.2229 (2)	0.4318 (3)	0.6983 (2)	0.0320 (4)	
C13	1.1449 (2)	0.3356 (3)	0.58707 (19)	0.0334 (4)	
H13	1.1873	0.2764	0.5402	0.040*	
C14	1.0033 (2)	0.3270 (3)	0.54499 (19)	0.0297 (4)	
H14	0.9482	0.2598	0.4689	0.036*	
C15	0.17419 (19)	0.4016 (3)	0.21639 (17)	0.0261 (4)	
C16	0.1142 (2)	0.3192 (3)	0.09396 (18)	0.0298 (4)	
H16	0.1714	0.2610	0.0615	0.036*	
C17	−0.0278 (2)	0.3218 (3)	0.01996 (18)	0.0315 (4)	
H17	−0.0688	0.2643	−0.0624	0.038*	
C18	−0.1079 (2)	0.4093 (3)	0.06815 (19)	0.0313 (4)	
C19	−0.0530 (2)	0.4919 (3)	0.1888 (2)	0.0328 (4)	
H19	−0.1112	0.5506	0.2200	0.039*	
C20	0.0880 (2)	0.4865 (3)	0.26212 (19)	0.0293 (4)	
H20	0.1275	0.5415	0.3454	0.035*	
C21	0.5127 (3)	0.5682 (4)	0.1057 (3)	0.0520 (7)	
H21A	0.4539	0.6705	0.1061	0.078*	
H21B	0.5206	0.5683	0.0241	0.078*	
H21C	0.6048	0.5817	0.1781	0.078*	

### Atomic displacement parameters (Å^2^)

**Table:**

	*U*^11^	*U*^22^	*U*^33^	*U*^12^	*U*^13^	*U*^23^
S1	0.0418 (3)	0.0475 (3)	0.0259 (2)	−0.0051 (2)	0.0186 (2)	−0.0059 (2)
F1	0.0243 (6)	0.0571 (9)	0.0646 (9)	0.0009 (6)	0.0185 (6)	−0.0050 (7)
F2	0.0245 (6)	0.0532 (9)	0.0509 (7)	0.0006 (6)	0.0073 (5)	0.0010 (6)
O1	0.0258 (6)	0.0405 (8)	0.0231 (6)	−0.0011 (6)	0.0119 (5)	−0.0019 (6)
C1	0.0279 (9)	0.0307 (10)	0.0228 (8)	−0.0028 (8)	0.0118 (7)	−0.0028 (7)
C2	0.0295 (9)	0.0258 (10)	0.0219 (8)	−0.0014 (7)	0.0125 (7)	−0.0011 (7)
C3	0.0281 (9)	0.0286 (10)	0.0256 (8)	−0.0024 (8)	0.0157 (7)	−0.0011 (8)
C4	0.0269 (9)	0.0207 (8)	0.0265 (8)	0.0014 (7)	0.0128 (7)	0.0010 (7)
C5	0.0266 (9)	0.0317 (10)	0.0228 (8)	0.0013 (8)	0.0083 (7)	0.0001 (8)
C6	0.0327 (10)	0.0372 (11)	0.0253 (9)	0.0010 (8)	0.0165 (8)	−0.0004 (8)
C7	0.0249 (8)	0.0317 (10)	0.0275 (9)	−0.0012 (8)	0.0124 (7)	−0.0004 (8)
C8	0.0303 (9)	0.0282 (10)	0.0233 (8)	−0.0024 (8)	0.0116 (7)	−0.0004 (7)
C9	0.0250 (9)	0.0217 (8)	0.0259 (8)	0.0020 (7)	0.0109 (7)	0.0054 (7)
C10	0.0286 (10)	0.0316 (10)	0.0290 (9)	0.0029 (8)	0.0146 (8)	−0.0024 (8)
C11	0.0298 (10)	0.0362 (11)	0.0321 (10)	0.0004 (9)	0.0091 (8)	−0.0049 (9)
C12	0.0240 (9)	0.0319 (10)	0.0417 (11)	0.0032 (8)	0.0159 (8)	0.0035 (9)
C13	0.0366 (10)	0.0317 (10)	0.0397 (10)	0.0057 (9)	0.0240 (9)	−0.0010 (9)
C14	0.0326 (10)	0.0265 (10)	0.0309 (9)	0.0016 (8)	0.0147 (8)	−0.0019 (8)
C15	0.0268 (9)	0.0246 (9)	0.0263 (8)	−0.0022 (8)	0.0112 (7)	0.0016 (7)
C16	0.0318 (10)	0.0287 (10)	0.0285 (9)	−0.0017 (8)	0.0128 (8)	−0.0009 (8)
C17	0.0346 (11)	0.0302 (11)	0.0238 (9)	−0.0029 (8)	0.0075 (8)	−0.0007 (8)
C18	0.0249 (9)	0.0289 (10)	0.0337 (9)	−0.0012 (8)	0.0071 (8)	0.0059 (8)
C19	0.0316 (11)	0.0305 (10)	0.0397 (11)	0.0022 (8)	0.0188 (9)	0.0003 (9)
C20	0.0341 (11)	0.0259 (9)	0.0262 (9)	−0.0020 (8)	0.0117 (8)	−0.0017 (7)
C21	0.0554 (16)	0.0591 (17)	0.0478 (14)	−0.0053 (12)	0.0286 (13)	0.0144 (12)

### Geometric parameters (Å, °)

**Table:**

S1—C1	1.7527 (18)	C10—C11	1.379 (3)
S1—C21	1.798 (3)	C10—H10	0.9500
F1—C12	1.354 (2)	C11—C12	1.375 (3)
F2—C18	1.355 (2)	C11—H11	0.9500
O1—C7	1.374 (2)	C12—C13	1.375 (3)
O1—C8	1.383 (2)	C13—C14	1.384 (3)
C1—C8	1.354 (3)	C13—H13	0.9500
C1—C2	1.444 (2)	C14—H14	0.9500
C2—C7	1.387 (2)	C15—C20	1.398 (3)
C2—C3	1.396 (2)	C15—C16	1.402 (3)
C3—C4	1.392 (2)	C16—C17	1.384 (3)
C3—H3	0.9500	C16—H16	0.9500
C4—C5	1.420 (3)	C17—C18	1.372 (3)
C4—C9	1.493 (2)	C17—H17	0.9500
C5—C6	1.373 (3)	C18—C19	1.385 (3)
C5—H5	0.9500	C19—C20	1.375 (3)
C6—C7	1.385 (3)	C19—H19	0.9500
C6—H6	0.9500	C20—H20	0.9500
C8—C15	1.461 (3)	C21—H21A	0.9800
C9—C14	1.395 (3)	C21—H21B	0.9800
C9—C10	1.395 (3)	C21—H21C	0.9800
			
C1—S1—C21	101.24 (12)	C10—C11—H11	120.9
C7—O1—C8	106.02 (14)	F1—C12—C13	118.83 (18)
C8—C1—C2	106.74 (15)	F1—C12—C11	118.70 (19)
C8—C1—S1	127.83 (14)	C13—C12—C11	122.47 (18)
C2—C1—S1	125.39 (14)	C12—C13—C14	118.42 (18)
C7—C2—C3	119.62 (16)	C12—C13—H13	120.8
C7—C2—C1	105.38 (15)	C14—C13—H13	120.8
C3—C2—C1	134.99 (16)	C13—C14—C9	121.27 (18)
C4—C3—C2	118.87 (16)	C13—C14—H14	119.4
C4—C3—H3	120.6	C9—C14—H14	119.4
C2—C3—H3	120.6	C20—C15—C16	118.72 (17)
C3—C4—C5	119.24 (17)	C20—C15—C8	119.79 (17)
C3—C4—C9	120.40 (16)	C16—C15—C8	121.49 (17)
C5—C4—C9	120.36 (16)	C17—C16—C15	120.55 (18)
C6—C5—C4	122.47 (17)	C17—C16—H16	119.7
C6—C5—H5	118.8	C15—C16—H16	119.7
C4—C5—H5	118.8	C18—C17—C16	118.58 (18)
C5—C6—C7	116.47 (17)	C18—C17—H17	120.7
C5—C6—H6	121.8	C16—C17—H17	120.7
C7—C6—H6	121.8	F2—C18—C17	118.40 (18)
O1—C7—C6	126.02 (17)	F2—C18—C19	118.85 (19)
O1—C7—C2	110.67 (15)	C17—C18—C19	122.75 (18)
C6—C7—C2	123.31 (17)	C20—C19—C18	118.17 (19)
C1—C8—O1	111.18 (15)	C20—C19—H19	120.9
C1—C8—C15	134.39 (17)	C18—C19—H19	120.9
O1—C8—C15	114.42 (15)	C19—C20—C15	121.21 (18)
C14—C9—C10	117.96 (17)	C19—C20—H20	119.4
C14—C9—C4	121.93 (16)	C15—C20—H20	119.4
C10—C9—C4	120.11 (16)	S1—C21—H21A	109.5
C11—C10—C9	121.60 (18)	S1—C21—H21B	109.5
C11—C10—H10	119.2	H21A—C21—H21B	109.5
C9—C10—H10	119.2	S1—C21—H21C	109.5
C12—C11—C10	118.28 (19)	H21A—C21—H21C	109.5
C12—C11—H11	120.9	H21B—C21—H21C	109.5
			
C21—S1—C1—C8	115.0 (2)	C5—C4—C9—C14	152.2 (2)
C21—S1—C1—C2	−67.4 (2)	C3—C4—C9—C10	151.49 (19)
C8—C1—C2—C7	0.0 (2)	C5—C4—C9—C10	−28.1 (3)
S1—C1—C2—C7	−178.03 (16)	C14—C9—C10—C11	0.9 (3)
C8—C1—C2—C3	−179.1 (2)	C4—C9—C10—C11	−178.74 (19)
S1—C1—C2—C3	2.9 (3)	C9—C10—C11—C12	−0.2 (3)
C7—C2—C3—C4	−0.8 (3)	C10—C11—C12—F1	179.42 (19)
C1—C2—C3—C4	178.1 (2)	C10—C11—C12—C13	−0.2 (3)
C2—C3—C4—C5	1.7 (3)	F1—C12—C13—C14	−179.68 (18)
C2—C3—C4—C9	−177.88 (18)	C11—C12—C13—C14	−0.1 (3)
C3—C4—C5—C6	−1.4 (3)	C12—C13—C14—C9	0.8 (3)
C9—C4—C5—C6	178.20 (19)	C10—C9—C14—C13	−1.2 (3)
C4—C5—C6—C7	0.1 (3)	C4—C9—C14—C13	178.46 (18)
C8—O1—C7—C6	179.7 (2)	C1—C8—C15—C20	−154.5 (2)
C8—O1—C7—C2	−0.3 (2)	O1—C8—C15—C20	24.2 (3)
C5—C6—C7—O1	−179.09 (19)	C1—C8—C15—C16	26.1 (3)
C5—C6—C7—C2	0.9 (3)	O1—C8—C15—C16	−155.20 (18)
C3—C2—C7—O1	179.44 (16)	C20—C15—C16—C17	−0.1 (3)
C1—C2—C7—O1	0.2 (2)	C8—C15—C16—C17	179.37 (17)
C3—C2—C7—C6	−0.5 (3)	C15—C16—C17—C18	1.1 (3)
C1—C2—C7—C6	−179.78 (19)	C16—C17—C18—F2	179.39 (17)
C2—C1—C8—O1	−0.1 (2)	C16—C17—C18—C19	−1.3 (3)
S1—C1—C8—O1	177.79 (15)	F2—C18—C19—C20	179.82 (18)
C2—C1—C8—C15	178.6 (2)	C17—C18—C19—C20	0.6 (3)
S1—C1—C8—C15	−3.5 (4)	C18—C19—C20—C15	0.5 (3)
C7—O1—C8—C1	0.3 (2)	C16—C15—C20—C19	−0.8 (3)
C7—O1—C8—C15	−178.75 (16)	C8—C15—C20—C19	179.82 (18)
C3—C4—C9—C14	−28.1 (3)		

### Hydrogen-bond geometry (Å, °)

**Table:**

Cg is the centroid of the C15–C20 4-fluorophenyl ring.

**Table:**

*D*—H···*A*	*D*—H	H···*A*	*D*···*A*	*D*—H···*A*
C6—H6···F1^i^	0.95	2.45	3.322 (2)	153
C10—H10···Cg^ii^	0.95	2.67	3.441 (2)	139
C17—H17···Cg^iii^	0.95	2.86	3.557 (2)	131

Symmetry codes: (i) *x*−1, *y*, *z*; (ii) −*x*+1, *y*+1/2, −*z*+1; (iii) −*x*, *y*−1/2, −*z*.
